# A Data-Driven Simulation of the Exposure Notification Cascade for Digital Contact Tracing of SARS-CoV-2 in Zurich, Switzerland

**DOI:** 10.1001/jamanetworkopen.2021.8184

**Published:** 2021-04-30

**Authors:** Dominik Menges, Hélène E. Aschmann, André Moser, Christian L. Althaus, Viktor von Wyl

**Affiliations:** 1 Epidemiology, Biostatistics and Prevention Institute, University of Zurich, Zurich, Switzerland; 2 CTU Bern, University of Bern, Bern, Switzerland; 3 Institute of Social and Preventive Medicine, University of Bern, Bern, Switzerland; 4 Institute for Implementation Science in Health Care, University of Zurich, Zurich, Switzerland

## Abstract

**Importance:**

Digital contact tracing (DCT) apps have been released in several countries to help interrupt SARS-CoV-2 transmission chains. However, the effect of DCT on pandemic mitigation remains to be demonstrated.

**Objective:**

To estimate key populations and performance indicators along the exposure notification cascade of the SwissCovid DCT app in a clearly defined regional and temporal context.

**Design, Setting, and Participants:**

This comparative effectiveness study was based on a simulation informed by measured data from issued quarantine recommendations and positive SARS-CoV-2 test results after DCT exposure notifications in the canton of Zurich. A stochastic model was developed to re-create the DCT notification cascade for Zurich. Population sizes at each cascade step were estimated using triangulation based on publicly available administrative and observational research data for the study duration from September 1 to October 31, 2020. The resultant estimates were checked for internal consistency and consistency with upstream or downstream estimates in the cascade. Stochastic sampling from data-informed parameter distributions was performed to explore the robustness of results. Subsequently, key performance indicators were evaluated to assess the potential contribution of DCT compared with manual contact tracing.

**Main Outcomes and Measures:**

Receiving a voluntary quarantine recommendation and/or a positive SARS-CoV-2 test result after exposure notification.

**Results:**

In September 2020, 537 app users received a positive SARS-CoV-2 test result in Zurich, 324 of whom received and entered an upload authorization code. This code triggered an app notification for an estimated 1374 (95% simulation interval [SI], 932-2586) proximity contacts and led to 722 information hotline calls, with an estimated 170 callers (95% SI, 154-186) receiving a quarantine recommendation. An estimated 939 (95% SI, 720-1127) notified app users underwent testing for SARS-CoV-2, of whom 30 (95% SI, 23-36) had positive results after an app notification. Key indicator evaluations revealed that the DCT app triggered quarantine recommendations for the equivalent of 5% of all exposed contacts placed in quarantine by manual contact tracing. For every 10.9 (95% SI, 7.6-15.6) upload authorization codes entered in the app, 1 contact had positive test results for SARS-CoV-2 after app notification. Longitudinal indicator analyses demonstrated bottlenecks in the notification cascade, because capacity limits were reached owing to an increased incidence of SARS-CoV-2 infection in October 2020.

**Conclusions and Relevance:**

In this simulation study of the notification cascade of the SwissCovid DCT app, receipt of exposure notifications was associated with quarantine recommendations and identification of SARS-CoV-2–positive cases. These findings in notified proximity contacts reflect important intermediary steps toward transmission prevention.

## Key Points

**Question:**

What is the possible contribution of digital contact tracing (DCT) apps to the interruption of SARS-CoV-2 transmission chains?

**Findings:**

This comparative effectiveness study found that the number of DCT app users receiving a quarantine recommendation corresponded to the equivalent of as many as 5% of all mandatory quarantined contacts identified by manual contact tracing in the canton of Zurich, Switzerland. Furthermore, approximately 1 in 11 notification triggers led to SARS-CoV-2 testing of an exposed proximity contact who subsequently had positive test results.

**Meaning:**

These findings suggest that DCT apps can make a relevant contribution to transmission prevention, with the potential to scale as more persons use the apps.

## Teaser

This data-informed simulation study estimates key populations and performance indicators along the exposure notification cascade of the SwissCovid digital contact tracing app in September and October 2020 in Zurich, Switzerland.

## Introduction

Switzerland was one of the first countries worldwide to release a digital contact tracing (DCT) app to complement manual contact tracing (MCT) in response to the COVID-19 pandemic.^1,2^ Digital contact tracing apps in many countries, including Germany, Italy, and Switzerland, follow the decentralized, privacy-preserving proximity-tracing blueprint (DP-3T) described by Troncoso et al.^3^ The DP-3T–based apps send out Bluetooth low-energy beacons (Bluetooth SIG) that include regularly changing, anonymous identification numbers, which are recorded and stored by other devices using the DP-3T app in the surrounding area (proximity contacts). App users with SARS-CoV-2–positive test results can trigger exposure notifications to their proximity contacts in a privacy-preserving manner by entering an upload authorization code in the DCT app.

In principle, DCT has several advantages over MCT. First, app users with SARS-CoV-2–positive test results do not have to rely on memory to recall proximity contacts. Second, once the app user enters the upload authorization code, notification happens almost in real time and simultaneously for all proximity contacts, without requiring human resources.^4^ Although detailed quantification and contextualization of the effects of DCT apps are warranted, early analyses based on routine monitoring data demonstrated proof-of-principle that DCT can work as intended. A study by Salathé et al^5^ showed that from July to September 2020, 65 persons in Switzerland had positive test results after an app notification (0.8% of 7842 reports containing information on reasons for testing). Furthermore, early population-level data were provided by studies from the Isle of Wight,^6^ and more recently from England and Wales.^7^ Based on statistical modeling of regional heterogeneity of app uptake and local incidence of SARS-CoV-2 infection, the latter study estimated the number of cases averted by successful app notification to range from 200 000 to 900 000 for November and December 2020.^7^

Focusing on the situation in Switzerland, our analysis had 2 aims. First, by combining data from different sources, we sought to describe the different steps in the notification cascade and to quantify the population in each step for a clearly defined regional and temporal context. Second, building on these results, we identified and estimated possible indicators to advance our understanding of the role of DCT vis-a-vis MCT. The analysis was conducted during a period of a marked increase in the incidence of SARS-CoV-2 infection, which brought MCT, testing laboratories, and health facilities in some areas to capacity limits. Therefore, our study also enabled a closer analysis of how potential health system bottlenecks may affect the notification cascade.

## Methods

Because this was a comparative effectiveness study based on aggregate data, it was exempt from requiring ethical approval under the Swiss Human Research Act. Informed consent was obtained from all participants in the COVID-19 Social Monitor^8^ and the Zurich SARS-CoV-2 Cohort^9^ studies contributing aggregated data for this analysis. The reporting and discussion of evidence follow the International Society for Pharmacoeconomics and Outcomes Research (ISPOR) reporting guideline where applicable.^10^

### Description of DCT Exposure Notification Cascade

**Figure 1.**
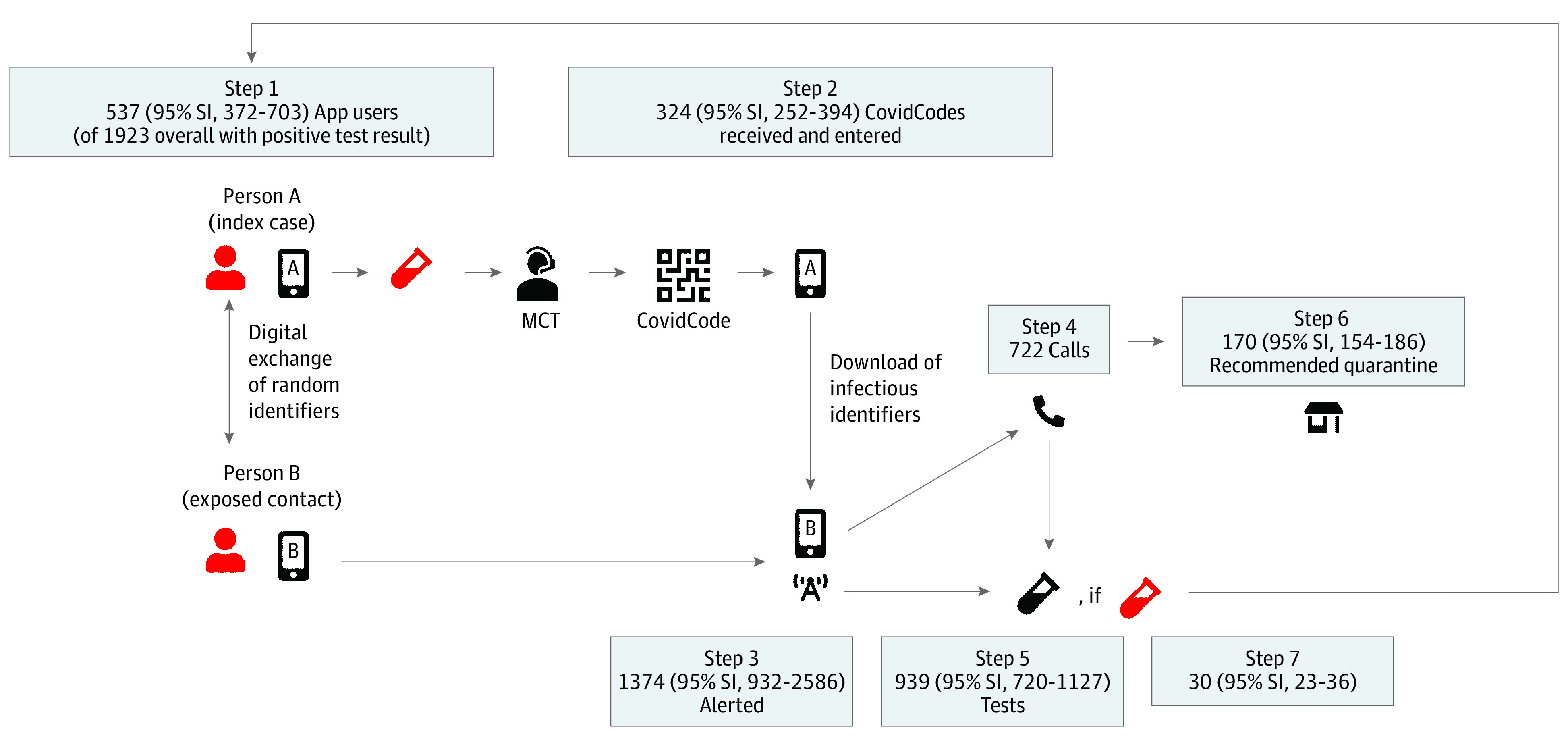
Conceptual Model of the Digital Contact Tracing Notification Cascade The notification cascade of the SwissCovid digital contact tracing app consists of the following steps: (1) An infected person A (index case) has a positive test result for SARS-CoV-2 (red), is referred to manual contact tracing (MCT), and (2) receives and uploads an authorization code (CovidCode) to warn other app users. Person B (exposed proximity contact) was in close proximity. (3) This person who receives the app notification has several options: (4) calling an information hotline (infoline; recommended option), (5) receiving a free test, and/or (6) voluntary quarantine. (7) Some persons will have a positive test result after app notification, who will then be placed in isolation by manual contact tracing. The different populations at each step were calculated using data triangulation and stochastic simulation, as described in Table 2. The numbers reflect simulation results for the canton of Zurich from September 1 to September 30, 2020 (first data column in Table 3); 95% simulation intervals (SIs) are calculated as 2.5th and 97.5th percentiles of 50 000 simulations.

A detailed description of the steps necessary for warning proximity contacts through DCT apps (the notification cascade)^11^ and parameterizations^12^ are provided elsewhere. Figure 1 illustrates the notification cascade in Switzerland. In brief, users of the SwissCovid DCT app with positive test results for SARS-CoV-2 must request an upload authorization code (“CovidCode”) from cantonal public health authorities (or, more recently, from physicians and testing centers). Entering the upload authorization code into the app will lead to a central server upload of all identification numbers that were broadcast within the time window of infectiousness. Other DCT apps periodically download the list of infectious identification numbers and match this list with the locally stored encounter identification numbers. The matching procedure takes proximity and time of the exposure into account and triggers an exposure notification if predefined thresholds (proximity of ≤1.5 m to an infected person for ≥15 minutes) are reached.

In Switzerland, notified app users are instructed to call an information hotline (hereinafter referred to as the infoline). The infoline staff will then determine whether a (voluntary) quarantine is warranted. Notified app users are also eligible for a free SARS-CoV-2 test, independent of an infoline call. Thus, the effectiveness of the app in stopping transmission chains strongly depends on the actions taken by notified app users to prevent further possible transmissions, such as entering quarantine.^2,4^

### Setting

**Figure 2.**
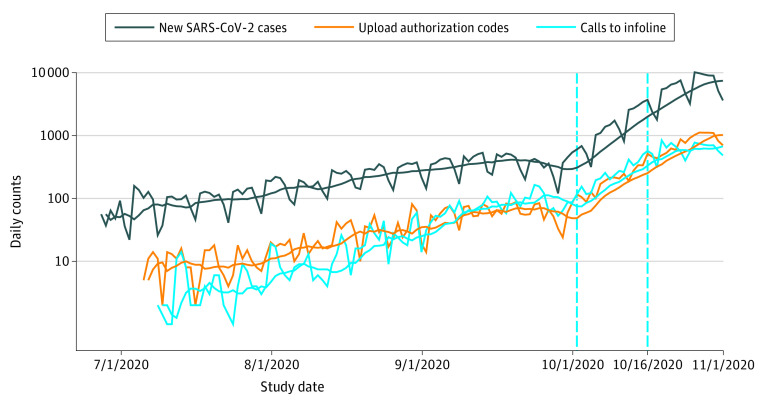
Evolution of Key Statistics in Switzerland for the Months of September and October 2020 Jagged lines are numbers from daily reporting, and smooth lines reflect 7-day moving mean numbers. Vertical blue lines mark cutoff dates for the different time strata used in the analysis. Infoline indicates information hotline.

We analyzed data collected from September 1 to October 31, 2020. As shown in Figure 2, the daily incidence of SARS-CoV-2 infection in Switzerland was relatively stable, at 300 to 600 new cases in September, followed by an increase in October to 10 000 cases and a decrease in November.^13^ Figure 2 further shows the number of entered upload authorization codes and infoline calls, which largely followed the trajectory of SARS-CoV-2 infection incidence. However, upload authorization codes and infoline call curves crossed in October, caused by high call volumes leading to a temporary overload at the infoline call center. There were also delays in issuing upload authorization codes for app users with positive test results, which have been documented by an increasing time from symptom onset to upload of authorization codes.^14^ Owing to these capacity issues, the analysis for the month of October was conducted for 2 separate periods (October 1-15 and 16-31, 2020). The primary study focus was the notification cascade in the canton of Zurich, which has 1.5 million inhabitants (compared with 8.6 million inhabitants in Switzerland overall). Zurich was selected owing to the availability of several relevant data sources from public health administration and research.^15,16^

### Data Sources

Key epidemiological data on use of the SwissCovid DCT app and indicators of app effectiveness were extracted from various sources (eMethods in the Supplement). Data are summarized in Table 1 for both Zurich and Switzerland overall.

**Table 1.** List of Input Parameters and Uncertainty Ranges

Parameter identifier	Parameter	Location						Source
		Zurich			Switzerland			
		September 1-30	October 1-15	October 16-31	September 1-30	October 1-15	October 16-31	
1A	Estimated No. in millions of active app users, No. (%)^a^	NA	NA	NA	1.6 (18.6)	1.6 (18.6)	1.8 (20.9)	Swiss Federal Office of Public Health^13^and Swiss Federal Office of Statistics^14^
1B	Measured No. of new SARS-CoV-2 cases	1923	3736	13 185	11 463	22 555	100 057	Swiss Federal Office of Public Health^13^
1C	App users among persons with positive test result, % (uncertainty range)^b^	27.9% (18.8-37.0)	28.0% (18.9-37.0)	28.9% (20.9-37.0)	27.9% (18.8%-37.0%)	28.0% (18.9-37.0)	28.9% (20.9%-37.0%)	von Wyl et al^16^
2A	No. of entered upload authorization codes for all of Switzerland	NA	NA	NA	1867	2916	12 453	Swiss Federal Office of Statistics^14^
2B	Logins into upload authorization code generation system from Zurich, % (4% point margin)	17.3 (13.3-21.3)	14.4 (10.4-18.4)	11.4 (7.4-15.4)	NA	NA	NA	NA
2B alternative	Fraction of new cases measured from Zurich, %	16.8	16.6	13.2	NA	NA	NA	Swiss Federal Office of Public Health^13^
3A	No. of measured calls to infoline after app notification	722	1289	3091	2430	3696	9889	Swiss Federal Office of Statistics^14^
3B	Notified users calling infoline estimated from the ZSAC, % (95% CI)	53.3 (26.6-78.7)	53.3 (26.6-78.7)	53.3 (26.6-78.7)	53.3 (26.6-78.7)	53.3 (26.6-78.7)	53.3 (26.6-78.7)	NA
4A	No. of calls to infoline measured after app notification^c^	722	1289	3091	2430	3696	9889	Swiss Federal Office of Statistics^14^
5A	Measured fraction of positive test results among all tests performed, %	3.2	9.4	15.8	3.6	10.9	23.8	Swiss Federal Office of Public Health^13^
6A	Calls leading to quarantine recommendation, % (10% margin)	23.5 (21.1-25.9)	21.7 (19.6-23.9)	23.0 (20.7-25.3)	21.5 (19.3-23.6)	19.8 (17.8-21.8)	20.3 (18.3-22.4)	NA
7A	No. of persons testing positive after app notification at midpoint (No. without correction; No. corrected for underreporting)^d^	29.5 (23; 36)	63.0 (52; 74)	75.5 (60; 93)	67.0 (52; 82)	121.5 (94; 149)	179.5 (139; 220)	NA

Abbreviations: NA, not applicable; ZSAC, Zurich SARS-CoV-2 Cohort Study.

^a^ Indicates fraction of Swiss population of 8.6 million.

^b^ Uncertainty range includes national mean based on parameter 1A; estimate from COVID-19 Social Monitor of 37%.

^c^ Same as parameter 3A.

^d^ Underreporting by 37% for all of Switzerland in September^5^ and likely higher in October 2020.

### Calculation Method

This study used a triangulation of measured data,^17^ combined with stochastic simulations to explore the robustness of estimates. The triangulation process followed the framework of Kaufmann et al.^18^ Key outcome measures were defined as described in Table 2, a list of possible administrative and research data sources was compiled, data sources were screened for relevant information, and suitable parameter data were extracted. Next, a schematic model for the notification cascade was defined, outlining the flow of users and information (Figure 1) analogous to the model presented elsewhere.^5^

**Table 2.** Calculation Process

Notification cascade step	Subpopulation to be estimated	Estimation process^a^
1	No. of app users with test results positive for SARS-CoV-2	No. of positive cases [1B] × fraction of app users in Swiss population [1A]
2	No. of uploaded authorization codes entered in app	No. of total upload authorization codes entered in Switzerland [2A] × percentage of logins into upload authorization code generation software by Zurich as a proxy for code generation [2B] (alternative: fraction of positive cases from Zurich [2B alt])
3	No. of persons notified by app	No. of infoline calls [4A]/percentage of persons calling the infoline among all persons notified by the app [3B]
4	No. of infoline calls related to app exposure notifications	No. of infoline calls [4A]
5	No. of tests performed after an app notification	No. of positive test results at app notification [7A]/percentage of positive tests among all tests performed [5A]^b^
6	No. of infoline calls leading to quarantine recommendation	No. of infoline calls [4A] × percentage of calls leading to quarantine recommendation [6A]
7	No. of positive test results after app notification	No. of positive test results where test was performed after app notification [7A]

^a^ Numbers in brackets refer to parameter identification in Table 1.

^b^ The overall test positivity rate is likely an underestimation of the true positivity for persons with digital proximity contact exposure.

For the first study aim, respective population sizes were calculated for each step in the notification cascade, based on measured data and estimated parameters (Table 2). In addition, the following complementary performance indicators were defined for the second study aim:

- Fraction of entered upload authorization codes per app users with positive SARS-CoV-2 results.
- Ratio of notified app users per entered upload authorization codes.
- Ratio of entered upload authorization codes per app users with positive test results after app notification.
- Ratio of the number of infoline callers receiving a quarantine recommendation to the number of all quarantined persons.
- Ratio of infoline calls per entered upload authorization codes.
- Ratio of persons with positive test results on app notification per all cases positive for SARS-CoV-2.

The triangulation was performed separately for the 3 different periods (all of September and the first and second halves of October) both for Zurich and for Switzerland overall. It centered around 2 key indicators, for which measured data were available: the number of infoline calls from Zurich (cascade step 4, parameter 4A) and the number of positive test results following an app notification in Zurich (step 7, parameter 7A; note that the measure is likely an underestimation^5^). The triangulation process consisted of a size estimation of the remaining 5 subpopulations in the notification cascade. For estimated parameters (Table 1), uncertainty ranges were either based on 95% CIs if parameters were derived from individual-level data or by adding a 4% to 10% margin around the best available data point if derived from aggregated data (with margin adjustment during the triangulation process described below).

Based on the parameters and uncertainty ranges, a stochastic simulation with 50 000 repetitions was performed, whereby all parameter values were drawn at random from uniform parameter distributions defined by prespecified parameter limits (Table 1). The estimated sizes of the 5 subpopulations in the cascade and the performance indicators were summarized as medians with 2.5th and 97.5th percentiles, including 95% of all estimates, for parameter estimates across all simulations (95% simulation interval [SI]). The simulation results were systematically checked for consistency based on the following criteria (in descending order of priority):

1. Is the estimate consistent with independently collected analogous data?
2. Does the estimate contradict other upstream or downstream estimates?
3. Are the estimates largely consistent with qualitative information if available?

In case of inconsistencies, the simulation was iteratively refined by adjusting the uncertainty ranges. Calculations were performed in R, version 3.6.2 (R Program for Statistical Computing).^19^

## Results

First, the population sizes at the different notification cascade steps were estimated. Figure 1 and eFigures 1 and 2 in the Supplement show subpopulation sizes of the individual cascade steps for Zurich in September 2020. Based on our simulations, 537 of 1923 persons (27.9%) with SARS-CoV-2–positive test results were app users and presumably received an upload authorization code. Of those, 324 (60.3%) entered an upload authorization code, which triggered exposure notifications in 1374 exposed proximity contacts. These notifications led to 722 persons calling the infoline, of whom 170 (23.5%) received a quarantine recommendation. Furthermore, 30 (95% SI, 23-36) app users had SARS-CoV-2–positive test results after receiving the app notification (3.2% of an estimated 939 [95% SI, 720-1127] notified persons undergoing testing).

Analogous calculations for the month of October and all of Switzerland are shown in Table 3 (upper half). As demonstrated in Figure 2 and Table 1 (second row), the number of SARS-CoV-2–positive cases in each period rose substantially in the first and second half of October in Zurich (from 1923 cases in September to 3736 and 13 185 cases in the first and second halves of October, respectively) and all of Switzerland (from 11 463 in September to 22 555 and 100 057 cases in the first and second halves of October, respectively). The absolute sizes of subpopulations in the notification cascade also grew markedly from September to October. The estimated number of entered upload authorization codes in Zurich increased from 324 (95% SI, 252-394) to 1426 (95% SI, 951-1896) from September to the second half of October; the number of infoline calls, from 722 to 3091; and the estimated number of quarantine recommendations, from 170 (95% SI, 154-186) to 711 (95% SI, 643-778).

**Table 3.** Quantification of the Digital Contact Tracing App Notification Cascade and Estimation of Monitoring Parameters

Outcome	Location, median (95% SI)					
	Zurich			Switzerland		
	September 1-30	October 1-15	October 16-31	September 1-30	October 1-15	October 16-31
Steps in notification cascade						
1: No. of app users among persons with positive test result	537 (372-703)	1042 (722-1365)	3818 (2809-4822)	3205 (2209-4191)	6296 (4365-8249)	28 931 (21 302-36 602)
2: No. of entered upload authorization codes	324 (252-394)	422 (310-532)	1426 (951-1896)	1867	2916	12 453
3: No. of notified app contacts	1374 (932-2586)	2457 (1665-4619)	5886 (3996-11 062)	4633 (3138-8707)	7020 (4773-13 244)	18 708 (12 772-35 445)
4: No. of calls to infoline related to app notification	722	1289	3091	2430	3696	9889
5: No. of tests after an app notification	939 (720-1127)	626 (509-743)	977 (406-1549)	3733 (1560-5906)	2378 (933-3814)	4255 (770-7764)
6: No. of calls to infoline leading to quarantine recommendation	170 (154-186)	280 (253-307)	711 (643-778)	522 (472-572)	733 (663-803)	2015 (1824-2207)
7: No. of persons with positive test result for SARS-CoV-2 after app notification	30 (23-36)	67 (53-82)	78 (61-94)	67 (53-82)	121 (95-148)	180 (141-218)
Other derived performance indicators						
Fraction of entered upload authorization codes per app user with positive SARS-CoV-2 test finding, %	60.3 (39.5-96.5)	40.4 (25.3-66.0)	37.3 (22.2-60.5)	58.3 (44.5-84.5)	46.3 (35.3-66.8)	43.0 (34.0-58.5)
Ratio of notified app users per entered upload authorization codes	4.3 (2.6-8.7)	6.0 (3.4-12.4)	4.3 (2.3-9.3)	2.5 (1.7-4.7)	2.4 (1.6-4.5)	1.5 (1.0-2.8)
Ratio of entered upload authorization codes per app user with positive test result after app notification	10.9 (7.6-15.6)	6.2 (4.2-9.3)	18.4 (11.2-28.3)	27.9 (22.8-35.2)	24.1 (19.7-30.7)	69.2 (57.1-88.3)
Ratio of the number of infoline callers receiving a quarantine recommendation per the number of all quarantined persons, %	5.1 (4.6-5.6)	3.5 (3.1-3.8)	3.1 (2.8-3.4)	2.0 (1.8-2.2)	1.7 (1.6-1.9)	2.0 (1.8-2.2)
Ratio of number of infoline calls per number of entered upload authorization codes (all of Switzerland)	NA	NA	NA	1.54	1.60	1.01
Ratio of persons with positive test result after app notification per all SARS-CoV-2 positive cases, %	1.6 (1.2-1.9)	1.8 (1.4-2.2)	0.6 (0.5-0.7)	0.6 (0.5-0.7)	0.5 (0.4-0.7)	0.2 (0.1-0.2)

Abbreviations: NA, not applicable; SI simulation interval.

Additional analyses of complementary performance indicators aiming to compare and evaluate the changes in subpopulation sizes with respect to the dynamics of the pandemic suggest that the number of entered upload authorization codes did not grow proportionally to the number of new cases of SARS-CoV-2, as shown in the lower half of Table 3. In Zurich, the fraction of entered upload authorization codes per app user with positive findings for SARS-CoV-2 changed from 60.3% (95% SI, 39.5%-96.5%) in September to 40.4% (95% SI, 25.3%-66.0%) in the first half of October and 37.3% (95% SI, 22.2%-60.5%) in the second half of October. The corresponding numbers for all of Switzerland were 58.3% (95% SI, 44.5%-84.5%) in September, 46.3% (95% SI, 35.3%-66.8%) in the first half of October, and 43.0% (95% SI, 34.0%-58.5%) in the second half of October.

Longitudinal indicator changes were also observed for the ratio of the number of infoline callers receiving a quarantine recommendation to the number of all quarantined persons, which declined from 5.1% (95% SI, 4.6%-5.6%) to 3.1% (95% SI, 2.8%-3.4%) in Zurich, whereas the nationwide ratio for Switzerland remained stable at 2.0% (95% SI, 1.8%-2.2%). Similarly, the ratio of app users with positive test results after an app notification to all persons with new positive test results changed from 1.6% (95% SI, 1.2%-1.9%) to 0.6% (95% SI, 0.5%-0.7%) in Zurich and from 0.6% (95% SI, 0.5%-0.7%) to 0.2% (95% SI, 0.1%-0.2%) in Switzerland. Similar dynamics were mirrored by the ratio of entered upload authorization codes per app users with positive test results on app notification. In Zurich, for every 10.9 (95% SI, 7.6-15.6) entered upload authorization codes, there was 1 positive test result after notification in September. This ratio decreased to 6.2 (95% SI, 4.2-9.3) in the first half of October, then increased to 18.4 (95% SI, 11.2-35.2) in the second half of October. The nationwide ratio for Switzerland showed a similar dynamic, but the nationwide ratios were 3 to 4 times higher than the ratios for Zurich (27.9 [95% SI, 22.8-35.2] entered upload authorization codes per detected case in September compared with 69.2 [95% SI, 57.1-88.3] in the second half of October).

## Discussion

### Main Findings

The present study provides a first estimation of the contribution of the DCT app to mitigating SARS-CoV-2 transmission in the canton of Zurich. During a period of relatively stable SARS-CoV-2 infection incidence in September 2020, we found that app notifications may have contributed to actions to prevent further viral transmission in 30 infected persons who underwent testing for SARS-CoV-2. Furthermore, we estimated that app notifications led to quarantine recommendations in 170 exposed contact persons. Overall, this estimate implies that the app could have led, at most, to a 5% increase of persons entering quarantine. However, the effort to identify these persons likely was less labor and resource intensive for DCT than for MCT. Of note, the data from Zurich suggest an above-average performance of the app notification cascade when compared with DCT indicators for all of Switzerland. Therefore, the results of our study suggest that DCT may have a role as a complementary measure to identify cases of infection and mitigate the spread of SARS-CoV-2.

The month of September was followed by a 20- to 30-fold increase in the incidence of SARS-CoV-2 infection by the end of October, which led to temporary capacity issues for MCT, infoline call centers, and the generation of upload authorization codes. Moreover, only 2 of 3 generated upload authorization codes are eventually entered by users in a timely manner.^5^ These cascade bottlenecks were also reflected by a deterioration of most outcome indicators monitored by our study. Nevertheless, the rising incidence of SARS-CoV-2 infection also had positive consequences for the adaptation and process efficiency of the DCT technology. From mid-August to mid-October 2020, the number of active app users in Switzerland plateaued at 1.6 million. Renewed public appeals by health authorities for using the app resulted in 200 000 additional users by the end of October.^14^ Moreover, important bottlenecks in the notification cascade, most especially the capacity of the infoline and the process for issuing upload authorization codes, were identified as a result of the increased incidence of infection. This led to the swift implementation of specific measures to improve DCT processes, such as technical solutions to handle higher call volumes and to automatize upload authorization code generation, as well as introducing the possibility for any physician and testing center to issue upload authorization codes if the cantonal health authorities are unable to respond in a timely manner.

Furthermore, cantonal public health authorities scaled up MCT by hiring more personnel and automated different steps in contact tracing through an online form for index cases and close contacts. Although the effects of these changes became fully visible after the conclusion of our analysis, some indicators from official statistics already suggested a recovery in the notification cascade performance. By November, the nationwide mean time from symptom onset to upload authorization codes entry decreased to approximately 4 days after a mean of 5 days in late October.^14^ Furthermore, owing to the introduction of rapid antigen testing at easily accessible sites (eg, including 40 pharmacies in Zurich), this interval is expected to have decreased further. These examples underscore the need for a continuous, comprehensive evaluation of the full DCT notification cascade, but also for a fast adjustment of processes and increase in resources in case of capacity bottlenecks. In addition, increased automation of the different steps in the notification cascade may help to achieve better performance. For example, DCT systems in Germany and Belgium have automated the registration and communication of SARS-CoV-2 test results or the upload of temporary exposure keys of infected persons, while still obtaining explicit app user consent for these steps.^20,21^ Similar optimizations are underway in Zurich.

### Strengths and Limitations

To our knowledge, our study is one of first systematic attempts to longitudinally quantify and evaluate the performance of the DCT app notification cascade. Our study is based on the premise that DCT apps represent a complex intervention that depends on the actions of app users and other players within a health system. A media analysis from Switzerland^22^ previously reported on implementation challenges for DCT, including delays in the provision of upload authorization codes to app users with positive test results. Our study confirms these observations and presents indicators for a more targeted monitoring of specific procedures along the notification cascade that pose potential bottlenecks.

The calculation methods and indicators used in this study for monitoring DCT performance do not require individual-level data and are thus fully compatible with the privacy-preserving design and philosophy of DP-3T. However, this is also a limitation. For example, the increased incidence of SARS-CoV-2 infections in the second half of October also affected the data collection procedures for monitoring statistics. For example, the reporting of the reasons for testing (such as a DCT app notification), which primarily relies on physicians diagnosing the infection, was already incomplete in September but nearly stopped toward the end of October 2020. This underreporting also may have led to the underestimation of some indicators in our analysis (eg, the number of persons with positive test results after app notification). However, we partly accounted for this issue through the performance of stochastic analyses and the presentation of uncertainty ranges.

Furthermore, some of the parameter estimates used in our calculations were derived from studies with limited sample sizes and follow-up (eg, the Zurich SARS-CoV-2 Cohort Study). Other parameters were only available on a national level, which may not reflect canton-specific differences adequately (eg, process efficiency of upload authorization code provision or MCT). These imprecisions are also reflected by the relatively wide uncertainty ranges of some parameters. However, we found no indications for systematic biases (eTable in the Supplement). To obtain more precise results involving fewer assumptions, more granular and more regionally differentiated data from ongoing research studies are essential.

## Conclusions

By evaluating the population at each step of the DCT notification cascade for Zurich and all of Switzerland, this simulation study provides one of the first estimations, to our knowledge, of the contributions of DCT apps to mitigating the pandemic. Our data suggest that the number of app-notified persons receiving a quarantine recommendation corresponds to the equivalent of as many as 5% of all mandatory quarantined contacts identified by MCT. Furthermore, we estimate that 1 in 10.9 persons who entered upload authorization codes led to SARS-CoV-2 testing of an exposed proximity contact who subsequently received a positive test result. Promoting use of the app, increasing automation of the DCT notification cascade, and connecting rapid antigen testing with DCT—while maintaining the privacy-preserving and voluntary nature of DCT—could further enhance the speed of the notification cascade and increase compliance of exposure-notified app users.

## Notes

Supplement. **eMethods.** Description of Data Sources **eTable.** Bias Assessment of Input Parameters **eFigure 1.** Bar Chart of Cascade Steps for SARS-CoV-2–Positive Index Cases **eFigure 2.** Bar Chart of Cascade Steps for Exposed Contacts **eReferences.**
