# Supplementary material for: A Data-Driven Simulation of the Exposure Notification Cascade for Digital Contact Tracing of SARS-CoV-2 in Zurich, Switzerland
Source: JAMA Netw Open. 2021 Apr 30;4(4):e218184. doi: 10.1001/jamanetworkopen.2021.8184 (PMC8087953; doi:10.1001/jamanetworkopen.2021.8184)
Supplement: Supplement. — eMethods. Description of Data Sources eTable. Bias Assessment of Input Parameters eFigure 1. Bar Chart of Cascade Steps for SARS-CoV-2–Positive Index Cases eFigure 2. Bar Chart of Cascade Steps for Exposed Contacts eReferences. [file jamanetwopen-e218184-s001.pdf]

Supplementary Online Content

Menges D, Aschmann HE, Moser A, Althaus CL, von Wyl V. A data-driven simulation of the exposure notification cascade for digital contact tracing of SARS-CoV-2 in Zurich, Switzerland. *JAMA Netw Open*. 2021;4(4):e218184. doi:10.1001/jamanetworkopen.2021.8184

eMethods. Description of Data Sources

eTable. Bias Assessment of Input Parameters

eFigure 1. Bar Chart of Cascade Steps for SARS-CoV-2–Positive Index Cases

eFigure 2. Bar Chart of Cascade Steps for Exposed Contacts

eReferences.

This supplementary material has been provided by the authors to give readers additional information about their work.

© 2021 Menges D et al. *JAMA Network Open*.

eMethods. Description of Data Sources

1. Federal Office of Public Health (FOPH). Data included the number of positive SARS-CoV-2 tests by age group and canton ***{1B}***.[1] The FOPH collects information on testing reasons for positive SARS-CoV-2 tests, including tests that noted a DCT app alert as a reason for testing ***{7A}***. These data were provided directly to the main author.
2. Federal Office of Statistics (FOS) and Federal Office of Information Technology,Systems and Telecomunication (FOITT). Publicly available data included the number of active DCT app users and the number of upload authorization codes ("CovidCodes").[2] The number of upload authorization code generations ***{2B}*** were provided directly to the main author.
3. Department of Health of the Canton of Zurich (DoH-ZH). The DoH-ZH compiles data on positive cases and operates MCT. Obtained data from this source include the number of positive cases in the Canton of Zurich ***{1B}***, the ratio of quarantined and isolated persons, the number of tests performed in Zurich and test positivity ***{5A}***.[3,4]
4. Medgate is a private company operating the national infoline, which users notified by the app are encouraged to call. It maintains statistics on number of infoline calls pertaining to app notifications ***{3A}*** and the percentage of infoline calls leading to quarantine recommendation ***{6A}***. While the number of calls is public [2], other information were provided directly to the main author.
5. COVID-19 Social Monitor is a longitudinal panel survey on COVID-19 in Switzerland, that includes between 1,500 to 1,700 respondents per wave.[5] The survey was started in March 2020, and 11 waves were completed by November 2020. It provided aggregated statistics on app usage ***{1A}*** and the percentage of app users calling the infoline after notification ***{3B}***. The main author had direct access to the data.
6. Zurich SARS-CoV-2 Cohort (ZSAC) Study is a longitudinal cohort study embedded in contact tracing of the Canton of Zurich [6] and provided aggregated data on the percentage of notified app users who called infoline ***{3B}*** and the percentage of index cases that were tested positive after an app notification ***{7A}***. The main author received the data directly.

© 2021 Menges D et al. *JAMA Network Open*.

eTable. Bias Assessment of Estimation Parameters

{1A}	Estimated number of active app users (fraction of Swiss population of 8.6 millions)	Measured, likely an underestimation because of technical limitations (e.g. persons temporarily switching of smartphones)
{1B}	Number of new SARS-CoV-2 cases (*measured*)	Measured, likely an underestimation of true case numbers
{1C}	Range percentage of app users among persons with positive test [national average based on *1A*; estimate from COVID-19 Social Monitor of 37%]	Estimation. Parameter range covers both conservative and more optimistic estimates
{2A}	Number of entered upload authorization codes *(measured for all of Switzerland)*	Measured.
{2B}	Percentage of logins into upload authorization code generation system from Zurich [±4%-point margin]	Measured, may be an over estimation if logins without generation of CovidCode
{2B alt.}	Fraction of new cases from the canton of Zurich (*measured*)	Measured by federal office of public health
{3A}	Number of calls to infoline after app notification *(measured)*	Measured by infoline
{3B}	Percentage notified users calling infoline, estimate from the ZSAC [95% confidence interval]	Estimate, may be prone to social desirability bias. Experiences from other countries suggest similar responder percentages (e.g. in the Netherlands, personal communication). Parameter sampling intervals are kept wide to reflect uncertainty.
{4A}	Number of calls to infoline after app notification (*measured*, same as *{3A}*)	Measured by infoline
{5A}	Fraction of positive test results among all tests performed (*measured*)	Measured by health authorities
{6A}	Percentage of calls leading to quarantine recommendation [±10% margin]	Measured by infoline. Margin added to reflect possible triage errors, recollection biases.
{7A}	Number of persons testing positive after app notification; midpoint [without correction; corrected for underreporting]	Estimated from a small fraction of all positive tests. But likely an underestimation. Data do not allow inference whether test were solely performed due to app notification or also due to other contributing factors, such as symptoms.

© 2021 Menges D et al. *JAMA Network Open*.

eFigure 1. Bar Chart of Cascade Steps for SARS-CoV-2–Positive Index Cases

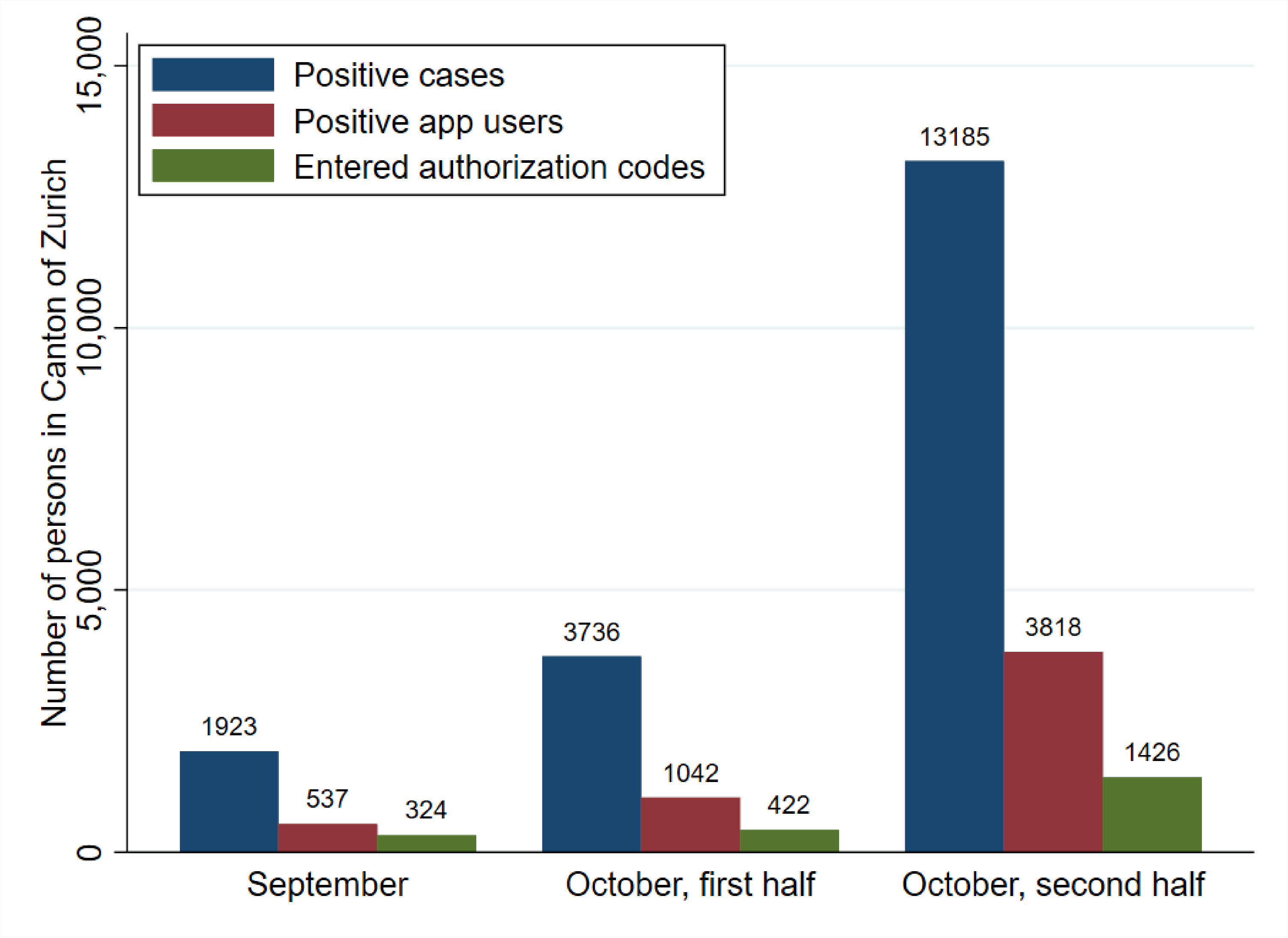

Corresponding to steps 1 and 2 in Figure 1 in main text.

© 2021 Menges D et al. *JAMA Network Open*.

eFigure 2. Bar Chart of Cascade Steps for Exposed Contacts

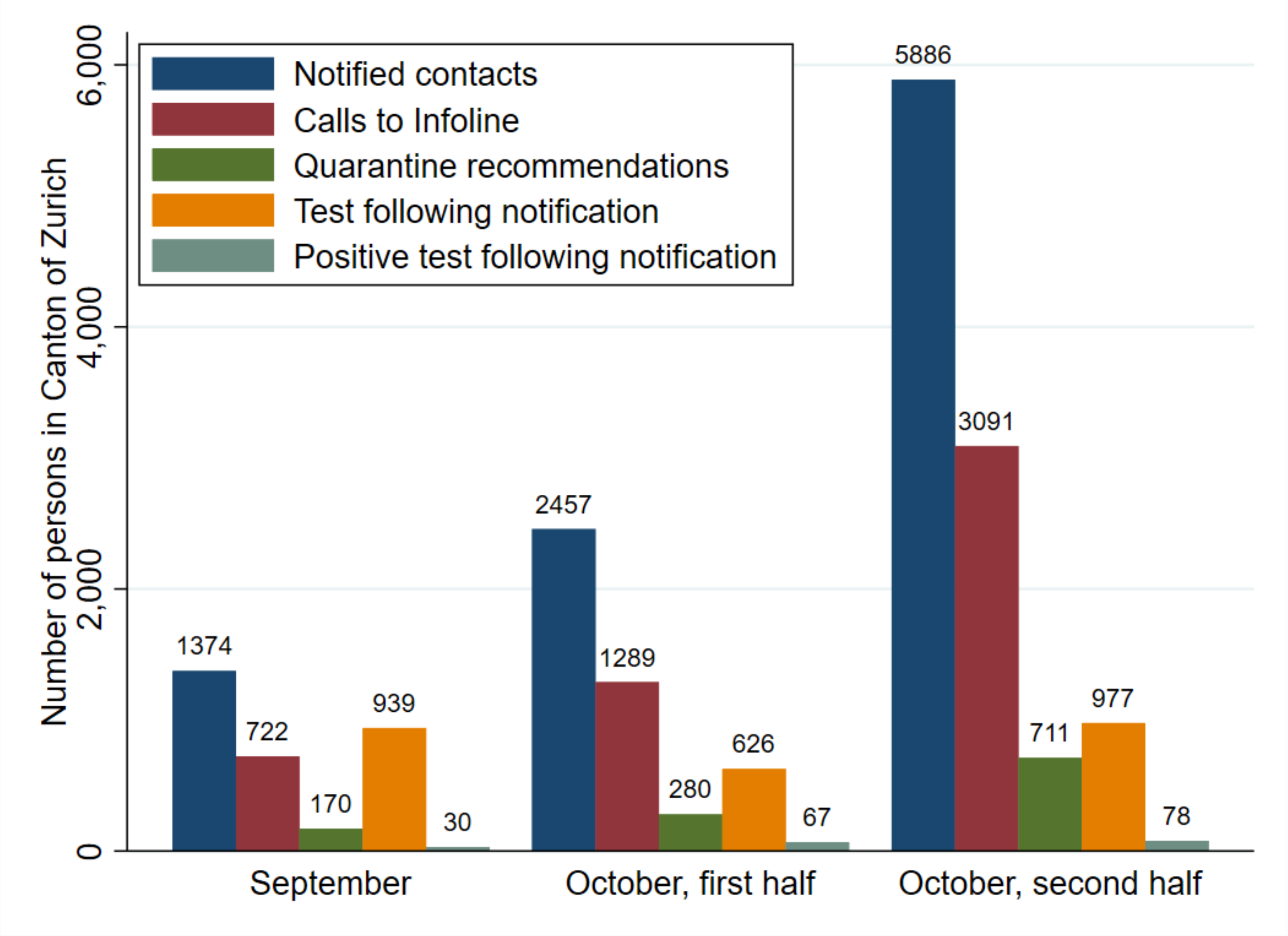

Corresponds to steps 3 to 7 in Figure 1 in main text

© 2021 Menges D et al. *JAMA Network Open*.

eReferences.

1. Swiss Federal Office of Public Health. Neues Coronavirus: Situation Schweiz. 2020; https://www.bag.admin.ch/bag/de/home/krankheiten/ausbrueche-epidemien-pandemien/aktuelle-ausbrueche-epidemien/novel-cov/situation-schweiz-und-international.html. Accessed 24.07.2020.
2. Swiss Federal Office of Statistics. SwissCovid App Monitoring. 2020. https://www.experimental.bfs.admin.ch/expstat/de/home/innovative-methoden/swisscovid-app-monitoring.html. Accessed 04.07.2020.
3. Department of Health of the Canton of Zurich. COVID_19 Fallzahlen Kanton Zürich. 2020; https://www.zh.ch/de/politik-staat/opendata.html?keyword=ogd#/details/671@gesundheitsdirektion-kanton-zuerich.
4. Department of Health of the Canton of Zurich. Zahlen & Fakten zu COVID-19. 2020; https://www.zh.ch/de/gesundheit/coronavirus/zahlen-fakten-covid-19.html?keyword=covid19#/home. Accessed 04.11.2020.
5. Moser A, Carlander M, Wieser S, Hämmig O, Puhan MA, Höglinger M. The COVID-19 Social Monitor longitudinal online panel: Real-time monitoring of social and public health consequences of the COVID-19 emergency in Switzerland. *PloS one.* 2020;15(11):e0242129.
6. Puhan MA, T.; Menges, D.; Koyuos, R.; Trkola, A.; Münz, C. Zurich Coronavirus Cohort: an observational study to determine long-term clinical outcomes and immune responses after coronavirus infection (COVID-19), assess the influence of virus genetics, and examine the spread of the coronavirus in the population of the Canton of Zurich, Switzerland. *ISRCTNregistry* 2020; http://www.isrctn.com/ISRCTN14990068. Accessed 04.11.2020.

© 2021 Menges D et al. *JAMA Network Open*.
